# Identifying Baicalin Concentration in Scutellaria Spray Drying Powder With Disturbed Terahertz Spectra Based on Gaussian Mixture Model

**DOI:** 10.1155/jamc/3858763

**Published:** 2024-11-20

**Authors:** Yizhang Li, Xiaodi Dong, Guiyun Cao, Yongbin Guo, Zhongmin Wang, Xiuwei Yang, Dongyue Han, Zhaoqing Meng

**Affiliations:** ^1^School of Electrical Engineering & Automation, Institute of Automation, Qilu University of Technology (Shandong Academy of Sciences), Key Laboratory of UWB & THz of Shandong Academy of Sciences, Jinan 250100, China; ^2^Traditional Chinese Medicine Research Institute, Shandong Hongjitang Pharmaceutical Group Co. Ltd, Jinan 250103, China

**Keywords:** baicalin, Gaussian mixture model, principal component analysis, scutellaria, terahertz spectroscopy

## Abstract

Baicalin concentration is concerned in manufacture of scutellaria spray drying powder as a traditional Chinese medicine, and the quality control based on high-performance liquid chromatography is inconvenience. In this study, terahertz time domain spectroscopy was employed to achieve quality control of scutellaria spray drying powder; however, an acute difficulty was found that terahertz spectra overlapped due to the disturbance in both content matrix and measurement error. In this study, similar terahertz spectra of scutellaria spray drying powder were classified with the help of Gaussian mixture model and built a classifier based on probability feature instead of spectral features conventionally employed in previous investigations. To explore the feasibility of GMM, principal component analysis was given, indicating that it is possible to train GMM with original features and proper principal components. Probable advantage of training GMM based on PCA feature was discussed and so it was with the capacity of the model to identify the linear combined spectra by comparing the performance of GMM and a decision tree model. Above all, the reason why GMM shows potential in the analysis of TCM terahertz spectra was illustrated by comparing the thought of discriminative model and generative model. This study implied that generative model may have natural advantage of overcoming the inherent disturbance of terahertz spectroscopy, which would be promising in future studies.

## 1. Introduction

Great progress has been made in terahertz spectroscopy years ago to characterize pharmaceutical molecules and the notable absorptions are generally attributed to hydrogen bond, selective vibration, and rotation of molecules [1–3]. The convenience and low cost in obtaining terahertz spectrum makes it competitive in characterizing the structure of compounds compared to chromatography. It is believed that prominent features related to certain molecular structures constitutes the basis of material identification, and thus, the materials with high chemical consistence and distinctive peaks are preferred for investigations [4, 5]. As a contrast, the overall response of compounds or mixtures at terahertz band is now attracting attentions from researchers as the identifications based on machine learning springs up which does not necessitate quantum chemistry calculations to reveal any energy level or molecular orbit, facilitating multidisciplinary studies by means of data mining. Complex and diverse algorithms make it possible to identify similar samples on condition that the potential feature is fully learned [6–10]. Nevertheless, the distribution complexity of terahertz curves due to both sample components and test environment requires further study using various models.

Scutellaria is a kind of traditional Chinese medicine that works for clearing heat and detoxicating in medical practices [11–14]. Baicalin is intensively concerned as its active component that has been proved antibacterial, diuretic, anti-inflammatory, cholesterol-lowering, antithrombus, and asthma relief [15, 16]. To take medicine in convenience, spray drying powder is made from scutellaria herbs whose baicalin concentration varies and the final curative effect is ensured by lowest limit of baicalin according to Chinese Pharmacopoeia. Conventionally, chromatography is employed to verify if the final product accords with the quality requirement. However, the close loop of chromatography measurement and empirical adjustment to raw material and technological parameters is considered time-consuming and expensive. It is possible to characterize scutellaria quality rapidly with terahertz spectroscopy, while the terahertz spectra with insufficient reproducibility challenge the accuracy of model. In particular, the nominal concentration (by chromatography) of the qualified spray drying powder and the unqualified one is just a bit away from the regulated limit in this task, which results from the mismatch between technological parameters and raw materials. As herbs contain plenty of chemical components whose ratio fluctuates, it is infeasible to measure terahertz spectrum of every component. To be worse, the slight difference in overall spectrum due to minor change in chemical constituents may be smoothed or offset by inherent error of terahertz source and detector. Thus, the observed spectra of spray drying powder are viewed as the disturbed ones from a standard since the concentration of active chemical components is the only known condition. It is critical to learn the slight difference by baicalin and deal with the spectral overlap by disturbing factors. It is critical to learn the slight difference by baicalin and deal with the spectral overlap by disturbing factors, which enables effective monitoring of scutellaria spray drying powder by using terahertz spectroscopy.

A number of investigators have committed to study the classification of mixture using terahertz spectroscopy and machine learning. Artificial neural networks (ANNs) and support vector machines (SVMs) are two kinds of representative and promising models in terahertz spectroscopy to predict sample information, whose structure varies under different backgrounds. Backpropagation neural networks (BPNNs), convolutional neural networks (CNNs), and recurrent neural networks (RNNs) are widely employed to classify terahertz spectra for samples in various groups, whose cell connection is specially designed to fit general spectra, 2D terahertz images, and time series data, respectively [17–21]. Li et al. reported the application of feedforward neural networks in gelatin identification [17], pointing out that the partition of dataset and random initiated parameters may have impact on model's performance. The phenomenon was related to the overfitting occurred to outlier samples, which may be neutralized by network cluster. Jiang investigated seed quality on terahertz wave images that were further digitized and used for training CNNs, resulting in a high model accuracy of 98.7% in quality identification [18]. Chen investigated classification of wheat grain varieties using terahertz spectrum subject to competitive adaptive reweighted sampling (CARS). The SVM, least square support vector machine (LS-SVM), BPNN, and CNN models were constructed using feature spectral data [19]. Gu et al. studied origin of panax notoginseng, suggesting that CNN-combined terahertz precision spectroscopy outperforms high-performance liquid chromatography due to wide band and nonlinear relationship [20]. Ren et al. employed long short-term memory neural network to correlate terahertz data with the reference data, making it possible to predict moisture content accurately [21]. The probable tendency can be forecasted that ANNs with more complex structures will be designed to achieve end-to-end accurate prediction, enabling multidisciplinary studies at a deeper level. Great potential of SVM models has also been exhibited in many studies targeting at nature resources, food, and biological samples. However, lots of efforts were made to pursue the best decision bound of SVM as the data-dependent hyperparameters were extremely important. Many SVM models are named by optimization algorithms or kernel function such as SSA-SVM (SVM optimized by sparrow search algorithm) [22], PSO-SVM (SVM optimized by particle swarm) [23], and GWO-SVM (SVM optimized by grey wolf optimizer) [24]. In feature extraction prior to building a discriminative model, methods including singular value decomposition (SVD) [25], principal component analysis (PCA) [26], wavelet analysis (WA) [27], variational mode decomposition (VMD) [28], and composite multiscale entropy (CMSE) [29] were widely concerned. It is implied by many reports that the supervised algorithms based on deep features of terahertz spectrum or images facilitate rapid and accurate identification of mixtures. As an example, Yan conducted SVD, nonnegative matrix factorization (NMF), and self-modeling mixture analysis (SMMA) to extract componential THz spectra [25, 30]. An overview of machine learning techniques can be found in Helal's work [30], which involves common techniques for terahertz curve processing.

**Figure 1:**
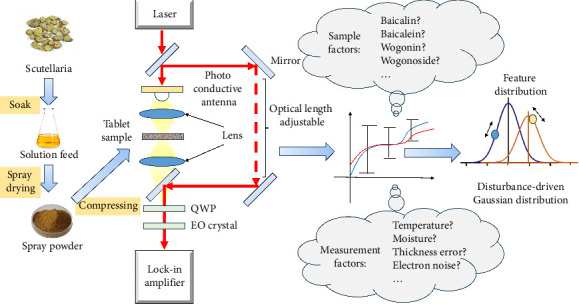
Visualization of the investigation thought.

Presently, the end-to-end prediction is preferred in classification missions where the different labels do not suggest regular changes in chemical components. However, the general constituents of qualified spray drying powder and unqualified ones are almost same. Despite fluctuation in various chemical components, the slight difference in baicalin concentration matters for both TCM regulators and the terahertz spectroscopy researchers. For this reason, it is necessary to adopt a model but a black box that describes the spectra distribution in real state. It is noticed that SVMs tend to determine hard borders and the method would be challenged if the input is unbalanced, badly contaminated by noise [31]. Therefore, we discuss the feasibility of applying Gaussian mixture model to analyze spray drying powders of scutellaria whose baicalin concentrations are almost the same but different in regulation sense. The thought of this study is illustrated by Figure 1, which shows the major steps to prepare sample, the basic light path of terahertz time domain spectroscopy, the puzzle we noticed, and the probable solution we came up with. A preprint concerning the study has previously been published [32]. This work implies that Gaussian mixture model may be an effective tool to deal with multivariate problem in terahertz spectrum analysis.

## 2. Methodology

Tablets of scutellaria drying powder with 13 mm diameter and various concentrations of baicalin were prepared in a metal die under a compressor at 20 MPa. The terahertz time domain spectroscopy was purchased from Zomega Corp, which consisted of a rotation delay line to support high sampling rate. The instrument supported temporal observation up to 110 ps and achieved spectral resolution superior to 10 GHz. To avoid interference from water vapor, the instrument was enclosed by a transparent close box, which was filled with dry air (humidity ≤ 3.0%). All the tests were carried out at room temperature, and every final spectrum for one sample is obtained by averaging 5000 spectra.

To calculate the optical parameter in terahertz band of interest, two tests were taken on sample (insert sample to test area) and reference (remove sample from the test area), respectively. The reference signal indicates time variant input terahertz pulse, while the sample signal indicates how sample corresponds to the input pulse in time domain. The optical parameters including refractive index and extinction coefficient are related to the chemical constituents of materials and the latter one is interpreted as the spectra in terahertz band. The difference of optical parameter within band underlies the identification capacity of mixtures, while the absorption at certain frequency is more concerned in studies of purities owing to hydrogen bond, collective vibration, or rotation of molecules. Optical parameter is calculated according to equations (1) and (2), where *ω* is the angular frequency, *ρ* is the amplitude ratio of sample and reference signal in frequency domain, *n* is the real part of complex refractive index, *ϕ* is the phase difference of sample and reference signal in frequency domain, *d* is the thickness of the sample, *κ* is the extinction coefficient, and *c* is the light speed of terahertz wave in vacuum, respectively. The detail of derivation and assumptions for sample are found in pilot work by Duvillaret, Garet, and Coutaz [33]. The frequency-dependent spectra indicated by *κ* are the original input as they are strongly influenced by molecular structure of the independent constituents and their combination. As a contrast, refractive index is not selected in many studies as the curves are flat and featureless for weak dispersion materials:

(1) $$\begin{array}{c} n=\frac{\phi c}{\omega d}+1, \end{array}$$

(2) $$\begin{array}{c} \kappa =\frac{c}{\omega d}\ln\left(\frac{4n\left(\omega \right)}{\rho \left(\omega \right){\left(1+n\left(\omega \right)\right)}^{2}}\right). \end{array}$$

PCA is employed to extract the feature for distinguishing samples with various concentrations and to eliminate the linear relationship between extinguishing coefficient values at different frequencies [34]. In short, the input of PCA model (*X*) is a matrix with *p* samples and *q* features, which is subject to zero-mean operation according to equation (3). Then, the covariance matrix divided by *p* is obtained according to equation (4); the eigen values and eigen vectors are calculated. Finally, project original input to the eigen vectors corresponding to bigger eigen values according to equation (5), resulting in output (*Y*) with lower dimensions. In equation (5), matrix *P* is included by eigen vectors with top *q*_0_ eigen values. The Gaussian mixture model can be established on the original curves or their downsampling in frequency domain. However, the spectrum values at different frequencies contain an implicit linear relationship, which is especially noticed for samples without remarkable absorptions. The upward trend of extinction coefficient is due to scattering of terahertz waves. In this case, PCA is commonly employed to reduce the number of variates, which may possibly have linear relationship. A comparison of Gaussian mixture models based on different inputs is found in the following equations:

(3) $$\begin{array}{c} X_{\left(p\times q\right)}=X-\text{mean}\left(X\right), \end{array}$$

(4) $$\begin{array}{c} M_{\text{covariance}}=\frac{1}{p}X^{T}X, \end{array}$$

(5) $$\begin{array}{c} Y=\text{XP}. \end{array}$$

Gaussian mixture model is employed in this study and it is assumed that samples are distributed following a combination of *K* multivariate normal distributions that vary in mean and variance [35–38]. In detail, it is considered that the terahertz frequency spectra for the qualified and the unqualified scutellaria spray drying powder follow two multivariate Gaussian distributions. It is also assumed that the difference in concentration of baicalin leads to the different mean while the disturbance in other constituents and measurement error result in variances. In this method, the prediction of unknown sample cannot be fulfilled until the key parameters regarding Gaussian distribution are estimated based on the given samples. Therefore, the probability distribution function (PDF) of one sample is denoted by equation (6) where the unknown parameter *θ* includes weight (*λ*_*k*_), mean (*μ*_*k*_), and variance (*σ*_*k*_); *k* is the index for certain multivariate normal distribution; and *N* denotes the multivariate normal distribution function. The analytic expression of the PDF for multivariate normal distribution function is described in equation (7), where *m* is the number of variates, Σ is the covariance matrix, and |Σ| is its determinant. The number of normal distributions, *k*, is a hyperparameter which is set to 2, suggesting the general distribution of samples in two groups. Unknown weights for *k*^th^ (1 ≤ *k* ≤ *K*, *k* is an integer) normal distribution are normalized, and thus, the sum of weights equals 1 (Σ*λ*_*k*_ = 1). The probability that one sample (indexed by *j*) follows certain normal distribution (indexed by *k*) is denoted by the equation (8):

(6) $$\begin{array}{c} P\left(x\left|\theta \right.\right)=\overset{K}{\underset{k=1}{\sum }}\lambda_{k}N\left({x,\mu }_{k},\sigma_{k}\right), \end{array}$$

(7) $$\begin{array}{c} N\left(x,\mu_{k},\sigma_{k}\right)=\frac{1}{{\left(2\pi \right)}^{\left(m/2\right)}{\left|\Sigma \right|}^{\left(1/2\right)}}e^{-\left({\left(x-\mu_{k}\right)}^{T}\Sigma^{-1}\left(x-\mu_{k}\right)/2\right)}, \end{array}$$

(8) $$\begin{array}{c} \gamma_{j,k}=\frac{\lambda_{k}N\left(x_{i}\left|\mu_{k}\right.,\sigma_{k}\right)}{\sum_{j=1}^{K}\lambda_{j}N\left(x_{i}\left|\mu_{j}\right.,\sigma_{j}\right)}. \end{array}$$

To train the model, expectation maximum (EM) algorithm is employed to figure out weight, mean, and variance for samples in two groups, which consists of two alternate steps in the limited times of iterations. In the first step of every iteration, the log-likelihood of GMM is derived, and to obtain the maximum of log-likelihood, the zero point of its derivation is figured correspondingly. In the second step of every iteration, the weight, mean, and variance of every normal are updated for next estimation according to equations (9)–(11). The parameters tend to converge after iterations, and thus, the presumed Gaussian distribution is obtained. To apply GMM model in this work, the number and the mean of *κ* for samples with different concentrations of baicalin are employed to initialize *λ*_*k*_ and *μ*_k_. In equation (9), *N* is the number of total samples, *N*_*k*_ is the number of samples whose distribution is governed by *k*^th^ Gaussian distributions, and *λ*_*k*_ is the weight. The main thought of EM algorithm is to conduct alternate calculations of hidden parameters (*λ*_*k*_) and the distribution parameters (*μ*_*k*_, *σ*_*k*_). As the maximum likelihood is pursued throughout the calculations, the results are increasingly compelling with the increasing of iterations:

(9) $$\begin{array}{c} \lambda_{k}=\frac{N_{k}}{N}, \end{array}$$

(10) $$\begin{array}{c} \mu_{k}=\frac{1}{N_{k}}\overset{N}{\underset{i=1}{\sum }}x_{i}, \end{array}$$

(11) $$\begin{array}{c} \sigma_{k}^{2}=\frac{1}{N_{k}}\overset{N}{\underset{i=1}{\sum }}{\left(x_{i}-\mu_{k}\right)}^{2}. \end{array}$$

## 3. Results and Discussion

**Figure 2:**
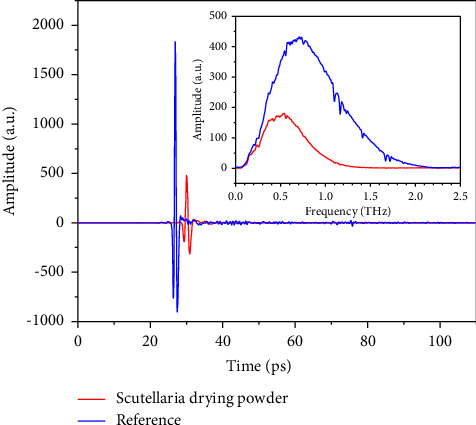
Typical terahertz signal for scutellaria spray drying powder and reference in TDS.

**Figure 3:**
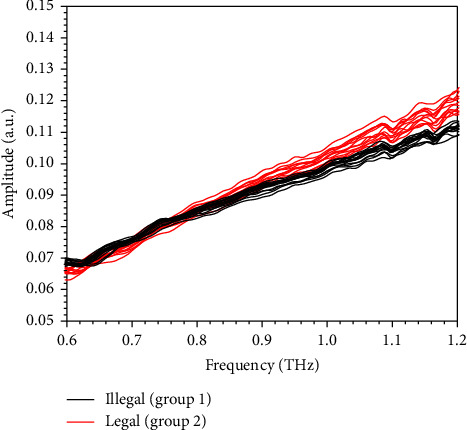
Distribution of terahertz extinction coefficients for scutellaria spray drying powder with different baicalin.

Typical terahertz spectrum of scutellaria spray drying powder and reference is shown in Figure 2. As seen in Figure 2, the amplitude of signal for scutellaria spray drying powder is notably attenuated from that of reference signal (∼1800) to ∼480, indicating the complicated interactions between multiple components and terahertz pulse. Correspondingly, the effective width for spectrum is reduced according to spectrum in frequency domain. Besides, the maximum amplitude for reference and sample signal is observed at 0.71 and 0.54 THz. It is verified by others that baicalin take possesses of unique absorbing features to identify itself from starch, which is commonly used in the industry of TCM [39]. However, the profile of baicalin in extinction coefficient of mixture is invisible since the overall absorbing from other components seems stronger and the superposition of spectral features is complex. Distribution of terahertz extinction coefficients for scutellaria spray drying powder with different baicalin is shown in Figure 3. In our study, the notable peaks for baicalin extinguishing are not observed according to Figure 3 and the most probable reason may be that the samples have light concentration of baicalin.

The main graph shows the time domain information, and the inset graph shows the frequency domain information.

According to the inset of Figure 2, band with convincing noise–signal ratio (NSR) is selected and the extinction coefficient is obtained according to equation (2). It is noticed that the terahertz spectra for spray drying powder with different baicalin concentration intervals lie in different regions at different frequencies. Generally, the group with higher baicalin concentration shows stronger absorption of terahertz wave from 0.6 to 1.2 THz. Nevertheless, a definite curve to identify curves with 100% accuracy is hard to determine owing to the overlap observed at any frequency of the concerned band. It is considered that both sample factors and measurement factors contribute to the phenomenon. Spray drying is the process of creating powder out of liquid through rapid drying in hot gas. For many thermally sensitive materials used in the food and pharmaceutical industries, it is the optimal drying method. However, the liquid or leachate of scutellaria contains various contents, whose fine control cannot be realized by setting time, temperature, and other engineer variables. As a result, the content matrix of spray drying powder is the combined effect of both raw material and technological factors. Thus, the content in spray drying powder fluctuated irregularly, leading to complex fluctuations in terahertz spectra. Besides, the investigator further transformed the samples from powder to tablets by compressing and obtained the terahertz spectra using THz-TDS. The error in measurement and the change in environment also attach disturbance to the final outcomes. In the study, we assume that the concentration of baicalin determines the center position of distribution while the change in residual constituents and measurement environment causes the dispersion of spectra. Therefore, it is very likely that the terahertz spectra for scutellaria spray powder follow Gaussian distribution, whose mean is mainly determined by a known factor (the concentration of baicalin). The spectral variations caused by both residual constituents and measurement environment are viewed as disturbance, which corresponds to variance and deviates single spectrum from the ideal ones. The ideal spectrum is also the spectrum for the spray drying powder with the average content matrix.

**Figure 4:**
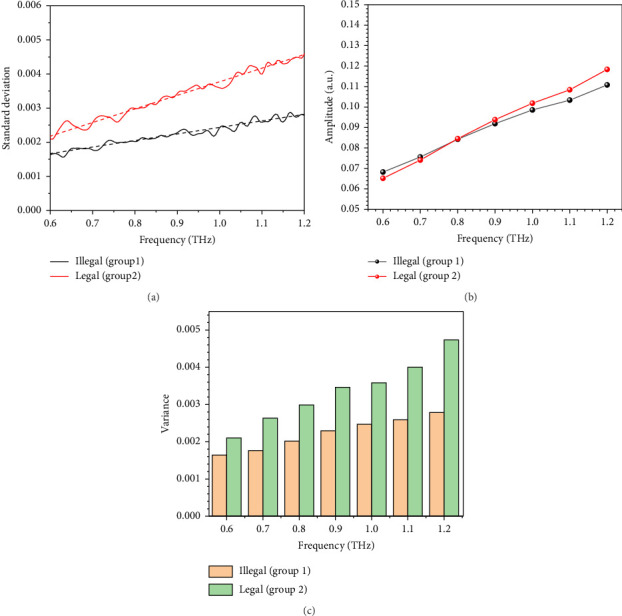
(a) Variance of samples for different groups in terahertz band of interest. (b) Mean of the selected features for samples calculated by GMM. (c) Variance of the selected features for samples calculated by GMM.

It is important to determine the dimensions of Gaussian distribution on account of both identifiability and calculation difficulty. Due to potential linear relationship between features, the positive definiteness of sigma matrix is not guaranteed on condition that the dimension is high and the robustness of GMM algorithm is challenged. The variance of samples for different groups in terahertz band of interest is shown in Figure 4(a). According to Figure 4(a), the variance of samples in two groups indicates approximate linear relationship (guided by dash lines) with the frequency despite of fluctuations and local maximum can be found at different frequencies with great difference in value. Therefore, we make use of 7 values of extinction coefficient at 0.6, 0.7, 0.8, 0.9, 1.0, 1.1, and 1.2 THz to train GMM. After training, the mean of samples with 7 features converges to the curve shown in Figure 4(b). As is seen, the converged mean of samples in 2 groups resembles the arithmetic mean of sample. As a comparison, the variance of the picked features is shown in Figure 4(c). According to Figure 4(c), the variance of the assumed distribution increases with the frequency, which is similar to the phenomenon reported in Figure 4(a). The results support the presumption that spectra for samples with different baicalin contents, but unknown other ingredients could be seen as curves following multivariate Gaussian distributions. It turns out that all 30 samples are correctly identified by comparing *γ*_*j*,*k*_, and the probability error is neglectable after 10 iterations.

**Figure 5:**
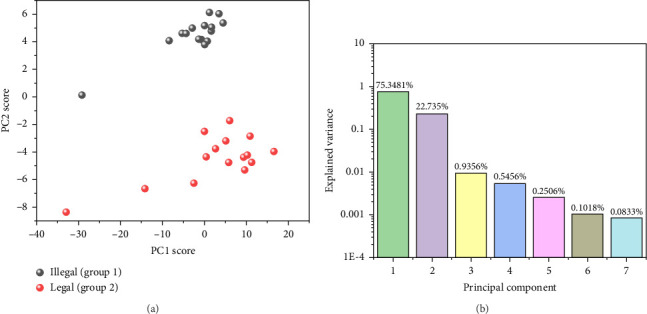
(a) The distribution of first two principal components for spray powder samples with various concentrations of baicalin. (b) The explained variance by different principal components.

**Figure 6:**
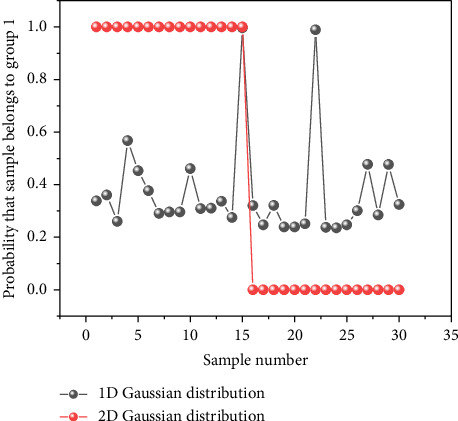
The performance of Gaussian mixture models in different dimensions.

The outcome of PCA is found in Figure 5 including the score distribution and the explained variance. It is found in Figure 5(a) that the top 2 principal components for most samples, but few outliers aggregate to two identifiable clusters. In addition, the explained variance of principal component decreases dramatically from 75.34% to 0.08% according to Figure 5(b). Considering the information provided by original 7 features and 7 principal components is equivalent, we assume that building GMM based on principal component is also possible. However, as first several principal components explain the approximate distribution of spectra which are conventionally employed in previous studies, we tried to train GMM models with the least number of principal components and the performance of different models is compared in Figure 6. As is seen, the result from GMM based on the first principal component is not as good as the expectation that the first 15 samples have *γ* close to 1 and the next 15 samples have *γ* close to 0. The result based on principal component indicates that the value of (max(*σ*_1_, *σ*_2_)/|*μ*_1_ − *μ*_2_|) is large, which goes against the accurate identification of labels. In the special case that *σ*_1_ equals *σ*_2_, the distribution of sample is symmetry with respect to 0.5∗(*μ*_1_ + *μ*_2_) according to the PDF. Correspondingly, a simple hard classifier is built according to the mean of points in two groups. In another special case that (max(*σ*_1_, *σ*_2_)/|*μ*_1_ − *μ*_2_|) is very small, a linear classifier with high accuracy is easy to build. In other words, disturbance attached to the optimal spectrum does not have negative effects on identifying samples. It is very likely that samples in two groups have notable differences in composition or the samples have minor differences, but the difference within the group is neglectable. As a comparison, when the combination of the first and the second principal component is employed, the results turn much more satisfactory and the deviation of value to the expected reaches 10^−3^ below. The results suggest that to assume the distribution of 2-dimensional principal component scores as 2-dimensional Gaussian distribution is more reasonable than to assume the distribution of first principal component as 1-dimensional Gaussian distribution. Besides, the iteration starts from the mean of scores, and thus, outliers have larger impacts if only one dimension is considered. The possible advantage of building GMM with principal component lies that PCA changes the range of feature, which may reduce the round-off error after iterations. To be noticed, the distributional correlation of original feature and PCA feature is not studied rigorously. We just report the feasibility of extracting GMM feature posterior to PCA feature but do not prove that normal distribution of original features results in normal distribution of its PCA features. In addition, it is not convincing to employ too many principal component features since the contribution of principal component drops dramatically according to their variance. As a result, the multivariate Gaussian distribution would tilt to normal distribution with fewer dimensions although more information is considered during training.

**Figure 7:**
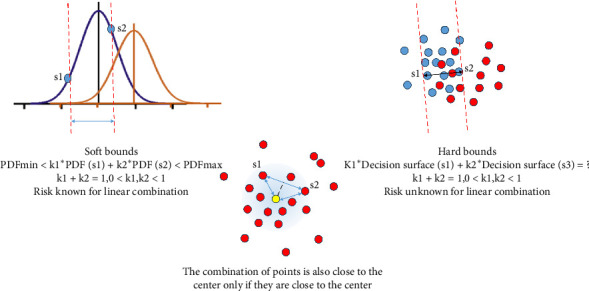
The inherent feature of GMM that outperforms discriminative models.

The advantage of using GMM to deal with spectra probably lies that it reduces the sensitivity of selecting proper principal components for some kinds of models as the linear combination of original feature is implicitly considered to learn multivariate Gaussian distribution though they are not involved in computation to avoid the problem of losing positive definiteness of sigma matrix. To verify the view, we build up a linear combined dataset to validate GMM model and a decision tree model trained with 2-dimensional principal components (maximal split is over the number of samples and the split criterion is Gini's diversity index), which both performs 100% accuracy for the 30 samples. Linear combination of spectral feature is accepted as the physical presumptions for the Beer–Lambert law that also works in terahertz band. By building the dataset, the content matrix made up by numerous unknown ingredients is reorganized as any two of 15 pseudo-independent spectra which are similar. The performance of GMM maintains satisfactory for any two-element linear combination of spectra, whereas the accuracy for the decision tree falls from 100% to 90.48% for the Group 1. Above all, the difference between two models reflects the difference in the thought of discriminative model and generative model in Figure 7. For discriminative models commonly studied, a definite decision bound is of more interest to terahertz spectroscopy researchers than the distribution of samples. However, due to outliers or inherent neighboring distribution, a definite hard decision bound is difficult to obtain. Because of the variation in the content matrix of spray drying powder as well as uncertainty in measurement, the actual spectra for two groups lie in a region that overlap to some degree (shown in Figure 7 left). Thus, we consider increasing the uncertainty tolerance to the extinction coefficient in terahertz band by assuming multivariate Gaussian distributions. In this study, *γ*_*j*,*k*_ is employed as an index to describe the probability that any sample belongs to Group 1 or Group 2, which is calculated by considering latent variables. The prediction risk for linear combination of any two trained samples can be estimated if the distribution condition is known. Intuitively, the linear combination of two points which lie at the neighborhood of the mean will also lie at the neighborhood of the mean in space of higher dimension (shown in Figure 7 middle). Above all, it is probable that the linear combination of outliers falls at the neighborhood of the mean, because the outliers may drag the spectral features to the opposite directions. That would be a vivid description of the deeper reason of GMM to overcome inherent disturbance in studies of TCM based on terahertz spectrum.

**Figure 8:**
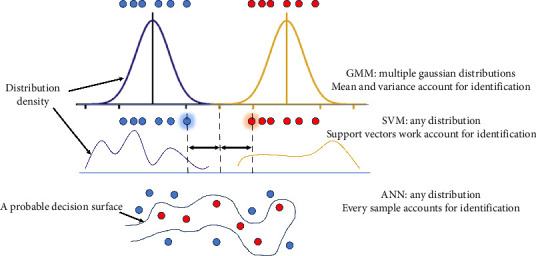
Comparison of GMM and current mainstream models employed to identify terahertz spectra.

The difference in classifying samples by GMM, SVM, and ANN is illustrated in Figure 8. For GMM, the distribution of sample is important and the predicted label is related to the local distribution density in feature space. The rationality of assuming Gaussian distribution underlies the good prediction on condition that the observed values of terahertz spectra are disturbed randomly by estimable factors. In this case, it is likely to recover real distribution from given samples and probability is employed to predict unknown sample. In SVM, classifier is built on special samples which guarantees minimum structure risk. If the points for different groups intertwine in distribution, the selecting of support vectors may change with the observations. In ANN, outstanding fitting ability of model leads to complex decision surface, and therefore, overfitting is common on condition that the features of original input do not cluster. In other words, GMM does not exceed conventional models in all tasks, but it truly has advantages for some tasks where the distribution of samples assembles Gaussian distribution due to specific constitutional reason. As a comparison, SVM (Gaussian kernel) is also trained with the extinction coefficients at 7 frequencies. It is found that the accuracy reached 93.3% for training set. As for the test set, however, the linear combination of samples in the same group does not always lead to the same label as expected. For combination of qualified (legal) samples and unqualified (illegal) samples, the prediction accuracy is 100% and 95.56%, respectively. However, if the manual kernel scale is reduced from 11 to 0.66, the training accuracy reaches 96.7%. For combination of qualified (legal) samples and unqualified (illegal) samples, the prediction accuracy turns to 91.64% and 100%, respectively. It is suggested that the adjustment of hyperparameters may have influence on the decision surface; however, such problem does not exist for GMM in this study as GMM tolerates overlap to some extent and it is not necessary to find a new feature space using a kernel function.

## 4. Conclusions

In this study, the baicalin concentration within spray powder of scutellaria is identified using terahertz optical parameter based on machine learning. In detail, PCA and Gaussian mixture component are employed to analyze disturbance in spectrum coupled to the concentration fluctuation of interest. Distribution up to 7 dimensions could be achieved using EM algorithm, which lead to mean and variance that resembles statistics based on real samples. The result of PCA indicates that assuming 2-dimensional multivariate Gaussian distribution based on first two principal components is more reasonable than to assume 1-dimensional Gaussian distribution solely based on the first principal component. GMM is appropriate for the missions including TCM in this study as it concerns about inherent disturbance, reducing the sensitivity of feature selecting in building classifier. Such view is verified by a dataset involving two-element linear combination of the trained samples, which is both employed in GMM and a typical decision tree model for comparison. The inner reason of GMM to overcome disturbance is discussed, indicating that GMM may play a role in analyzing complex terahertz spectra under various backgrounds.

## Data Availability

The data that support the findings of this study are available from the corresponding author upon reasonable request.
